# Anatomical Bases of the Temporal Muscle Trigger Points

**DOI:** 10.1155/2024/6641346

**Published:** 2024-02-24

**Authors:** Luis Carlos Fernandez Garrido, Giulianna Simonetti, Samir Omar Saleh, Flávio Hojaij, Mauro Andrade, Alfredo Luiz Jacomo, Flavia Emi Akamatsu

**Affiliations:** ^1^Department of Surgery, Laboratory of Medical Research—Division of Human Structural Topography, Faculty of Medicine of the University of São Paulo (FMUSP), São Paulo, SP, Brazil; ^2^Department of Surgery Medicine, Laboratory of Medical Research, FMUSP, São Paulo, SP, Brazil

## Abstract

**Method:**

Temporal muscles of 14 adult cadavers were studied. The muscle bellies were divided into six areas, three superior (1.2 and 3) and three inferior areas (4, 5, and 6) lower, according to a Cartesian plane to analyze and describe the entry points of the branches of the deep temporal nerves into the muscle. The branching distribution was analyzed using Poisson log-linear tests with Bonferroni post hoc tests for comparison between groups (sextants) (*p* < 0.05).

**Results:**

Deep temporal nerve entry points were found in the temporal muscle in all areas. Most of the branches were observed in areas 2 and 5, which coincide with the muscle fibers responsible for mandible elevation and related to the previously described MTPs. Fewer branches were found in areas 1 and 6, where contraction produces mandible retraction.

**Conclusion:**

There is an anatomical correlation between the branching pattern of the deep temporal nerve and temporal muscle trigger points. Adequate knowledge of the innervation of the temporal muscle may help elucidate the pathophysiology of myofascial syndromes and provide a rational basis for interventional or conservative approaches and help surgeons avoid iatrogenic lesions to the deep temporal nerve lesion.

## 1. Introduction

Myofascial pain syndrome is present in 45% of patients diagnosed with temporomandibular dysfunction (TMD), which affects 38% to 75% of Europeans and 30% of Asians, with a higher incidence in young adults aged between 30 and 40 years and a predominant incidence in women [1, 2]. One study on the prevalence of orofacial pain in 250 nursing students identified MTPs in the mastication muscles, and 43% of these MTPs were located in the temporal muscles [3]. Other studies showed that up to two-thirds of patients with temporomandibular dysfunction reported pain in the temporal muscle [4, 5]. Myofascial pain syndrome (MPS) is the most common cause of musculoskeletal pain, and it affects three-quarters of the population worldwide [6]. It may be diagnosed in acute or chronic forms. MPS is characterized by a set of clinical findings, including hyperirritable areas called trigger points (TPs), which relate closely to clinical manifestations and pathophysiology [7]. TPs are distinctive features of MPS and are used in the differential diagnosis of other causes of muscle pain, such as inflammatory disorders and fibromyalgia. One or more tender points in the muscle may be due to trauma, excessive muscle use, psychological stress, and systemic diseases [8]. Travell and Simons were the first to describe myofascial trigger points (MTPs) based on clinical observations, which were later supported by additional studies of MTPs as a source of musculoskeletal pain [9, 10]. The pathophysiology of MPS is associated with dysfunction of the motor plate area [9–12]. Sensory symptoms (e.g., referred pain, hyperalgesia, and dysesthesia), motor (movement limitation), and autonomic symptoms (e.g., runny nose, tearing, salivation, changes in skin temperature, sweating, piloerection, proprioceptive disorders, and erythema of the underlying skin) are present in MPS and caused by MTPs [13].

Even though dry needling has been reported to treat temporomandibular joint dysfunction, some studies offer poor scientific evidence to support its widespread use. Moreover, its mechanism of action remains unclear, and no relevant references are made to the anatomical nerve branching, which may play a significant role for this therapeutic approach [14].

The temporal muscle originates at the temporal fossa, immediately superior to the inferior temporal line. Its insertion is on the coronoid process of the mandible and the front margin of the mandible ramus. The muscle is fan shaped. Its anterior fibers are directed vertically, and its posterior fibers horizontally cross the temporal fossa. The medium fibers have intermediate degrees of obliquity. The temporal muscle is innervated by the deep temporal nerve, which is a branch of the anterior trunk of the mandibular nerve, and it is the most powerful acting nerve on the temporomandibular joint. Its anterior fibers elevate the mandible, while their posterior portion primarily retracts the mandible [15].

Some reports on temporal muscle innervation have been published. However, no reference has been made to the branching distribution of the deep temporal nerve into the muscle belly [16, 17].

The present study investigated the detailed innervation of the temporal muscle in cadavers to provide an anatomical basis for clinically described MTPs. This additional information provides a better understanding of the pathophysiology of MPS and auxiliary mapping of the branching of the deep temporal nerve for conservative and interventional approaches.

## 2. Material and Methods

### 2.1. Ethical Aspects

The Ethics Committee of Medical School for the Analysis of Research Projects approved this study (protocol no. 4.013.660).

### 2.2. Anatomical Technique

**Figure 1:**
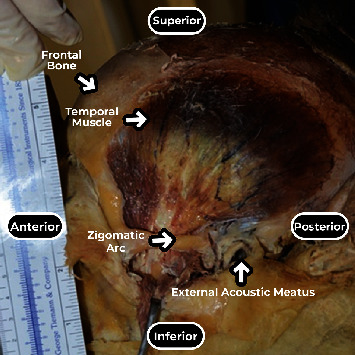
Lateral view of dissected left temporal muscle.

Twenty-eight temporal muscles from cadavers donated to the Discipline of Human Structural Topography of the Department of Surgery of the University of Faculty of Medicine were dissected to expose the branches of the deep temporal nerves and their points of entry into the temporal muscle. The 14 cadavers (6 males and 8 females) were previously fixed with a 4% phenolic acid and 0.5% formaldehyde solution. No specimens had deformities or previous manipulation of the temporal area. During dissection, the cadavers were placed in the supine position, and a median incision was made from the upper lip up and backward until the external occipital protuberance. The skin and subcutaneous tissue were reflected, and the superficial temporal fascia was exposed with the anterior, superior, and posterior auricular muscles. After resection of these structures, the deep temporal fascia and the underlying temporal muscle were thoroughly exposed (Figure 1).

### 2.3. Measurements of the Temporal Muscle and Delimitations of the Sextants

**Figure 2:**
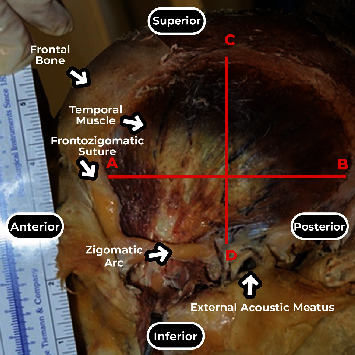
Lateral view of the left temporal muscle. Transverse (AB) and longitudinal (CD) measurements.

**Figure 3:**
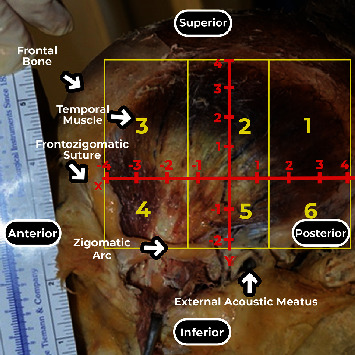
Lateral view of the left temporal muscle. Abscissa *X*, ordered *Y*, and separation in sextants (1, 2, 3, 4, 5, and 6).

Morphometric measurements of the muscle dimensions (transverse and longitudinal) were performed (Figure 2). We divided the entire area of the temporal muscle into six subareas to describe the branching pattern of the deep temporal nerves in the muscle, three superior (1, 2, and 3) and three inferior (4, 5, and 6), according to a Cartesian plane (Figure 3). These six subareas were based on two reference lines: a transversal line through the frontozygomatic suture to posterior border of temporal muscle (AB, axis *x*) and a second line perpendicular to the first, crossing the midpoint between the transversal line (CD, axis *y*) (Figure 2).

By convention, the following were adopted: the intersection of the axes as the origin and zero point, upper posterior quadrant with positive ordinate and abscissa, lower anterior quadrant with negative ordinate and abscissa, upper anterior quadrant with negative abscissa and positive ordered, and lower posterior quadrant with positive abscissa and negative ordered (Figure 3).

The data were grouped into categories, forming six areas of distribution to facilitate clinical correlation. The middle transverse line separated the upper and lower areas and was divided into three equally sized segments. On these three segments, perpendicular lines were drawn according to Figure 3.

From the dimensions of the temporal muscle, the penetration points of the branches of the deep temporal nerves in the temporal muscle were measured according to the muscle transverse and longitudinal diameters to delimit a Cartesian plane. This method was previously described by Akamatsu et al. to evaluate the location of the penetration points of the nerve branches in the muscle belly using anatomical dissection [18].

**Figure 4:**
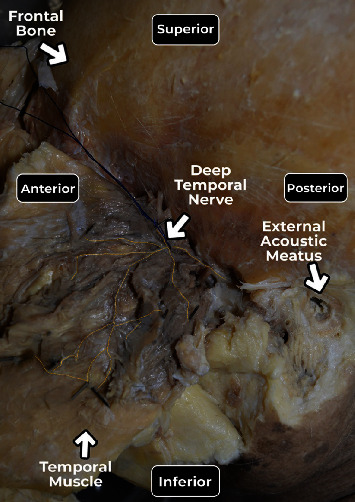
Lateral view showing the deep temporal nerve in the reflected left temporal muscle after removal of the zygomatic arch, yellow color marks the nerve until the entry points into the muscle and the blue wire separating the nerve.

After defining the sextants, the zygomatic arch was removed, and the temporal muscle was carefully retracted to expose the neurovascular bundles and identify the branching pattern of the deep temporal nerve (Figure 4).

**Figure 5:**
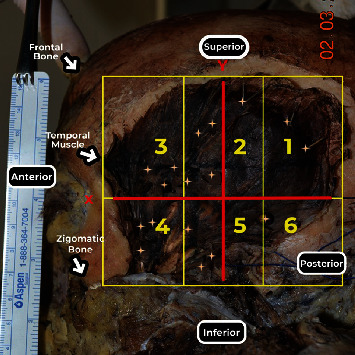
Lateral view of the left temporal muscle. Stars showing the intake of the branches of the deep temporal nerves into the muscle. Points in sextants.

The points of entry of the branches of the deep temporal nerve into the muscle were indicated with pins and documented photographically using a Nikon D52 camera (Nikon Corporation; Tokyo, Japan). Penetration points were measured in relation to the median longitudinal and transverse axes by a simple division of values and classified according to the numbered area from 1 to 6 (Figure 5).

### 2.4. Statistical Analysis

During the first four procedures, the maximal variability of the entry points of the temporal nerve in each area of interest selected was 1.88, which estimated that at least 2 more points could be found in the most innervated area compared to the least innervated area. A calculated sample of 14 cadavers was obtained with a power and confidence of 80 and 95%, respectively. As our findings demonstrate a variation wider than one entry point, our sample reflects the expected results from a larger population. It was considered for the 2-sided test calculation. For sex, muscles were described using summary measurements, and comparisons were made using Student's *t* tests [19]. Mann–Whitney tests were used to compare the total number of points in each individual muscle according to sex, and the paired Wilcoxon test [19] was used to compare the number of points between sides. The number of points related to each of the areas analyzed was described and compared between areas using generalized estimation equations with an interchangeable correlation matrix among sides and sextants using a Poisson marginal distribution and identity binding function [20], followed by multiple Bonferroni comparisons [21] to identify areas (1 to 6) that showed differences. Pearson's correlation method [22] was employed between anthropometric descriptors and the number of entry points of the deep temporal nerves in the temporal muscle belly, as the association between this points and the muscle measured dimensions. Analyses were performed using IBM-SPSS for Windows version 26.0 software (IBM Corp.; released 2019; IBM SPSS Statistics for Windows, version 26.0; Armonk, NY; IBM Corp.). The tests were performed with a significance level of 5% (*p* < 0.05).

## 3. Results

Fourteen cadavers were used, 8 females and 6 males. Thirteen were Caucasian, and one was Asian. Age ranged from 56 to 95 years (mean = 76.3 years), weight from 45 to 93 kg (mean = 60.16 kg), and height from 1.50 to 1.85 m (mean = 1.67 m) (Table 1).

**Figure 6:**
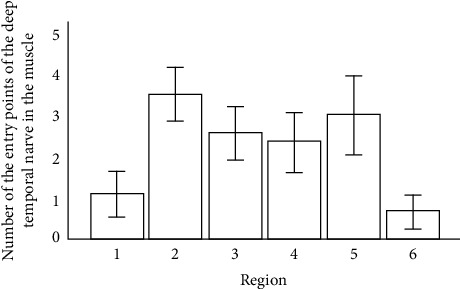
Entry points of the deep temporal nerves in the muscle belly. Grade points average on all sextants and standard deviation.

**Figure 7:**
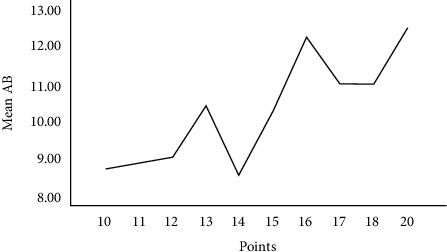
Correlation between the size of the transverse AB measurement and the number of nerve entry points into muscle.

The longitudinal and transversal dimensions of the right and left temporal muscles were compared, and dimensions differed between female and male cadavers, except for the longitudinal length of the left temporal muscle (*p* > 0.05) (Table 2). Entry points of the deep temporal nerves in the muscle belly were found in all sextants of all cadavers irrespective of gender and side (Figure 6). The number of entry points of the deep temporal nerves that penetrated the muscle belly showed significant differences between sextants (*p* < 0.001) (Table 3). Area 2 was the most innervated, with more entry of points, followed by sextant 5, but no significant differences were noted. Sextants 3 and 4 had fewer entry points than 2 and 5, but there was no significant difference. Sextants 1 and 6 showed fewer entry points (*p* < 0.05). There was no correlation between anthropometric data (age, gender, race, and BMI) and the number of entry points of the deep temporal nerves in the temporal muscle (Pearson's correlation) (Table 4). Regarding muscle measurements, we observed a positive correlation between the transversal length (AB) and the number of entry points of deep temporal nerves (*p* < 0.05) (Figure 7).

## 4. Discussion

**Figure 8:**
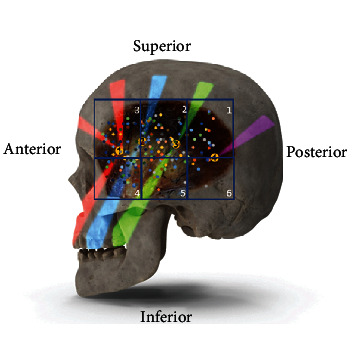
Trigger points 1, 2, 3, and 4, described by Travell and Simons [9], sites of reflex pain of each point in the colored bands. Sextants 1,2,3,4,5,6, with colored dots showing the intake of the branches of the deep temporal nerves into the muscle of the cadavers used in this study.

Some authors reported patterns of deep temporal nerve branching to the temporal muscle [16, 23]. Our work hypothesized that innervation of the temporal muscle was related to MTPs, which pain in the temporal muscle and facial region. Our group published studies of other muscles (e.g., trapezius, gluteus maximus, masseter, and hallux abductor muscles) and found a correlation between trigger points and the anatomical identification of nerve entry points [18, 24–26]. Travell and Simon described four trigger points in the temporal muscle and their respective areas of related pain [9]. Trigger point 1 is located at the anterior region of the muscle, trigger points 2 and 3 are located in the intermediate part of the muscle, and trigger point 4 is found on the posterior region of the muscle [9]. Trigger points 1, 2, and 3 are located at the musculotendinous junction and the posterior, and trigger point 4 is at the muscular belly [27] (Figure 8).

Studies on the distribution map of nerve branching to the trapezius muscle suggested that dysfunction of the innervation zone was responsible for the emergence of MTPs [28, 29]. This hypothesis is further supported by the physiopathological events following muscle injuries. Inflammatory mediators are released and activate nociceptors, and removal of acetylcholine from the synaptic cleft is decreased. Excess acetylcholine provokes hyperstimulation of postsynaptic receptors, which leads to the persistent contraction of muscle fibers that is a characteristic feature of MTPs [30, 31].

Previous studies described the presence of three branches of the deep temporal nerves: anterior, middle, and posterior branches [32, 33]. We also found three main branches that penetrated the anterior, middle, and posterior areas of the temporal muscle. There may be two or three deep temporal nerves [15, 34].

Happak et al. [35] studied facial muscles and demonstrated that the motor plates were located near the nerve entry points in the muscle. Our dissections found the highest number of penetrations of the deep temporal nerves into the belly of the temporal muscle in the region of nerve entry into the muscle.

Due to the fan-shaped distribution of its muscle fibers, the function of the temporal muscle varies according to the area of main contraction. One study used electromyography and demonstrated higher activity in the anterior region of the muscle during mastication compared to the posterior region [36].

Notably, the area where we found the highest number of entry points of the deep temporal nerve was the region with perpendicular fibers that is responsible for the elevation of the mandible [37] and the strongest contraction of the temporal muscle [36]. However, the posterior part of the muscle, which is responsible for mandible retraction and lateral movements due to its horizontal fibers, receives fewer branches of the nerve, which is likely because less strength is needed to perform these actions for the lateral and medial pterygoid muscles [36].

Although a large number of points were found in regions 3 and 4, these areas were less innervated than areas 2 and 5. This region contains perpendicular and oblique fibers, which contribute to the elevation of the mandible [15].

Our study observed that the transverse length of the muscle correlated with the number of nerve branches, which likely provide adequate nerve supply based on its shape [23, 35, 38].

Our initial hypothesis that trigger points of the temporal muscle corresponded to the branching pattern of the deep temporal nerves seems supported by our findings, provided that they correspond to the clinical location of MTPs described by Travell and Simons [9].

The anatomical basis of the myofascial trigger points was reviewed by Ziembicki, supporting the hypotheses of the correlation of the muscle entry points with the trigger point phenomenon [39]. He also pointed out the reproducibility and confidence level of our method which demonstrated the topographical superposition of the MTP with nerve branching to the muscles [40]. According to Ziembicki, nerve entry points are anatomical characteristics of the clinical presentation [39].

Therefore, we propose that an anatomical basis exists to justify myofascial pain disorders related to the temporal muscle, and we found similar results in other muscles. Also, these findings may represent the anatomical basis for percutaneous insertion of the needles as in dry needling approach to produce pain reduction and restore temporomandibular range of motion [41].

These findings may help practitioners in the management of this common source of chronic pain and its accompanying changes in quality of life [42].

## 5. Conclusion

The anatomical distribution of the branches of the deep temporal nerve to the temporal muscle corresponds to clinical MTPs. Its knowledge is an important tool for conservative or interventional therapies, and it is paramount to avoid iatrogenic lesions during surgical approaches. Anatomy elucidates the uncertain physiopathology of myofascial pain syndromes.

## Figures and Tables

**Table 1:** Description of the characteristics of the cadavers.

Variable	Description (*N* = 14)
Age (years)	
Average ± SD	76.30 ± 13.58
Median (min.; max.)	79.50 (56; 95)
Sex, *n* (%)	
Female	8 (57.10)
Male	6 (42.90)
Race, *n* (%)	
White	13 (92.90)
Yellow	1 (7.10)
Height (m)^∗^	
Average ± SD	1.67 ± 0.01
Median (min.; max.)	1.73 (1.50; 1.85)
Weight (kg)^∗^	
Average ± SD	60.16 ± 14.81
Median (min.; max.)	55 (45; 93)
BMI^∗^	
Average ± SD	22.02 ± 4.39
Median (min.; max.)	21.74 (15.9; 30.5)

Note: ^∗^12 cadavers had the information.

**Table 2:** Description of temporal muscle measurements by sex.

Variable	Sex		Total	*p*
	Female (*N* = 8)	Male (*N* = 6)	(*N* = 14)	
Right temporal muscle AB (cm)				
Average ± SD	8.25 ± 0.46	11.50 ± 1.67	9.64 ± 1.99	0.001^∗∗^
Median (min.; max.)	8.25 (7.50; 9.00)	11.25 (9.00; 14.0)	8.75 (7,5; 14,0)	
CD (cm)				
Average ± SD	7.62 ± 0.64	9.16 ± 1.21	8.28 ± 1.18	0.020^∗^
Median (min.; max.)	7.50 (7.00; 9.00)	9.50 (7.50; 10.50)	7.75 (7.00; 10.50)	
Left temporal muscle AB (cm)				
Average ± SD	8.93 ± 1.05	11.25 ± 2.16	9.92 ± 1.94	0.029^∗^
Median (min.; max.)	8.75 (8.00; 11.00)	11.50 (8.00; 14.50)	9.50 (8.00; 14.50)	
CD (cm)				
Average ± SD	7.85 ± 1.08	9.08 ± 1.24	8.37 ± 1.27	0.081
Median (min.; max.)	7.75 (6.50; 10.00)	9.25 (7.00; 10.50)	8.25 (6.50; 10.50)	

Note: Student's *t* test; ^∗^*p* < 0.05; ^∗∗^*p* < 0.001.

**Table 3:** Description of the number of points of entry of the deep temporal nerve branches according to sextant.

Sextant	Average ± SD	Median (min.; max.)	*p*
1	1.11 ± 1.42	1 (0; 6)	<0.001^∗∗^
2	3.50 ± 1.68	3 (0; 8)	
3	2.57 ± 1.77	2 (0; 8)	
4	2.36 ± 1.94	2 (0; 7)	
5	3.00 ± 2.46	3 (0; 8)	
6	0.68 ± 1.05	0 (0; 4)	

Note: ^∗∗^*p* < 0.001. Note: EEG with the Poisson distribution and identity binding function.

**Table 4:** Description of entry points of the deep temporal nerve branches in the temporal muscle according to sex.

Variable	Sex		Total	
	Female (*N* = 08)	Male (*N* = 06)	(*N* = 14)	*p*
Total temporal muscle				0.961
Average ± SD	25.25 ± 4.77	27.83 ± 4.02	26.36 ± 4.49	
Median (min.; max.)	24 (20; 35)	27.50 (23; 33)	25 (20; 35)	
Right temporal muscle				0.472
Average ± SD	12.25 ± 2.60	14.50 ± 3.20	13.21 ± 2.99	
Median (min.; max.)	11.50 (10; 18)	13.50 (12; 20)	12.00 (10; 20)	
Left temporal muscle				0.151
Average ± SD	12.63 ± 2.44	13.33 ± 1.63	12.93 ± 2.09	
Median (min.; max.)	11.50 (10; 17)	13 (11; 16)	13 (10; 17)	

Note: Mann–Whitney *U* test.

## Data Availability

The data used to support the findings of this study are included within the article, but if it is necessary to consult all the data of the table with each of measurements of entrance of nerves per quadrant of the corpse feet, they are available from the corresponding author upon request.
